# Histopathological Lesions Caused by a Digenean Trematode in a Pest Apple Snail, *Pomacea canaliculata*, in Its Native Geographic Distribution Area

**DOI:** 10.3390/ani14081191

**Published:** 2024-04-16

**Authors:** Lorena Evangelina Martinez, Carmen Gilardoni, Cintia Débora Medina, Florencia Cremonte, Jorge Alejandro Etchegoin

**Affiliations:** 1 Instituto de Investigaciones en Producción Sanidad y Ambiente (IIPROSAM), CONICET-Universidad Nacional de Mar del Plata, Centro de Asociación Simple CIC-PBA, Juan B. Justo 2550, Mar del Plata 7600, Argentina; lorenamlertora@gmail.com (L.E.M.); jetchego@mdp.edu.ar (J.A.E.); 2 Instituto de Biología de Organismos Marinos (CCT CONICET-CENPAT), Boulevard Brown 2915, Puerto Madryn 9120, Argentina; fcremont@gmail.com; 3 Instituto de Ciencias del Mar (ICM-CSIC), Passeig Marítim de la Barceloneta, 37–49, 08003 Barcelona, Spain; 4 Instituto de Diversidad y Evolución Austral (CCT CONICET-CENPAT), Boulevard Brown 2915, Puerto Madryn 9120, Argentina; cintia.d.medina@gmail.com

**Keywords:** mollusc, Ampullaridae, echinostomatid, histopathology, host response

## Abstract

**Simple Summary:**

The apple snail is one of the most dangerous invasive species in freshwater environments. Using molecular and morphological tools, we re-describe an echinostomatid digenean parasitizing snails from two sites in the Buenos Aires Province, Argentina. The two stages found (i.e., rediae and metacercariae) demonstrate that the apple snail acts as the first and second intermediate host in its life cycle. The prevalence of the parasite was higher at one of the sampling sites, probably because the birds bearing the adult stage are more abundant in that area. A histological study showed that this parasite quickly invades multiple organs of the snail, which is different from most digenean infections, which only infect the gonad and digestive glands. Heavy deterioration of female and male reproductive structures associated with the presence of the parasite was also observed, which indicates castration.

**Abstract:**

*Pomacea canaliculata* is one of the most dangerous invasive species. Morphological and molecular analyses have revealed that a digenean species belonging to the family Echinostomatidae parasitizes this snail at two sites in Buenos Aires Province, Argentina, South America. Molecular results confirmed that the species belongs to a genus closely related to *Patagifer*. Analysis of the 28S rDNA showed that the sequences of the rediae and metacercariae are identical, indicating that the apple snail acts as the first and second intermediate host. The cercariae may encyst as metacercaria inside the redia and also emerge and re-infect the same snail or another snail. The prevalence of digeneans was higher in one of the sampling locations (15.1% vs. 0.72%), probably because the bird species that acts as the definitive host is more abundant in that area. Histopathological examination showed that the parasite quickly invades multiple host organs (gills, intestines, albumen gland, lung, kidney, and mantle border) besides the gonad and digestive gland, as is usual in digeneans. In addition, the partial or total castration of snails was observed in cases of moderate and high infection intensity. In males, there was loss of integrity in testicular tubules, while in females, the replacement of ovarian tissue by rediae was found.

## 1. Introduction

The golden apple snail *Pomacea canaliculata* (Lamarck, 1822) (Ampullariidae, Caenogastropoda) is native to South America but has spread, colonizing several countries such as the United States, Spain, China, and Taiwan [1]. Furthermore, it has been expanding its original distribution in Argentina [2]. In fact, *P. canaliculata* is currently included in the most dangerous group of invasive freshwater snails; it causes habitat alterations, modifies benthic community structures, and decreases local diversity [3,4]. According to the IUCN, it is listed as one of the top one hundred worst invasive species [5]. 

In addition to being able to cause damage to local ecosystems, *P. canaliculata* may act as a host to several parasite species, including trematode digeneans [6,7]. These parasites have complex life cycles with at least two hosts, including free-living and parasitic stages. Intra-molluscan stages (sporocysts and/or rediae) proliferate asexually in the snail when it acts as the first intermediate host; they migrate through blood spaces until reaching the tissues, from which they obtain nutrients [8,9]. These intra-molluscan stages can cause histological damage and structural alterations that may include the castration of the snail host [10,11,12,13,14]. Beyond providing energy for multiplication, infecting the host’s gonad allows the parasites to improve conditions for survival without causing severe damage and to complete their life cycles [15,16,17]. The digenean parasite–host association shows high specificity, mainly at the level of the snail as its first intermediate host. In these cases, the parasite evades the host’s immune response by avoiding recognition and elimination (see [18]). This fact demonstrates that this strong association comes from a long co-evolutionary history [19]. 

To date, at least eight different species of digeneans have been reported to use the apple snail *P. canaliculata* as an intermediate host [7,20]. None of them have been identified by molecular studies, and histological studies have not been carried out to assess the damage they cause. In Los Talas, Buenos Aires Province, Martorelli [21] reported that the species *Echinostoma parcespinosum* Lutz, 1924 (Echinostomatidae) uses the apple snail as its first intermediate host in its native distribution area. In members of this genus, the rediae give rise to the swimming larval stage, cercariae, capable of emerging from the snail in search of a second intermediate host. These cercariae may re-infect the same snail host species, becoming an encysted metacercariae. The same species of digenean may have, as an alternative, an abbreviated life cycle, which implies that the cercariae do not leave the snail and become encysted metacercaria within the rediae. When the definitive host, usually a bird, feeds on the snail, the metacercariae mature sexually into an adult stage, completing the life cycle [8,9].

This work aimed to describe a digenean belonging to the family Echinostomatidae in the invasive apple snail, *Pomacea canaliculata*, in its native range distribution area by using morphological and molecular methods. Furthermore, via histology, we assessed the sites of infection and lesions inflicted on snail tissues.

## 2. Materials and Methods

### 2.1. Sample Collection and Morphological Description

Sampling was conducted during the summer period of 2022–2023; 67 individuals of *Pomacea canaliculata* were collected from a small lagoon in the Harbor Natural Reserve (R) (38°01′ S, 57°31′ W) and 137 snails from Los Padres Lagoon (LP) (37°56′ S, 57°44′ W), Buenos Aires Province, Argentina. Snails were collected by hand, searching the bottom of submerged and floating vegetation along a transect parallel to the shoreline. Specimens were kept in separate small freshwater containers under a 12-12 light–dark photoperiod for 48 h to stimulate the shedding of the digenean larvae (cercariae) and inspected twice daily under a stereomicroscope (Zeiss Stemi508, Zeiss, Jena, Germany) for emerged cercariae. The latter were stained with vital stain (neutral red or Nile blue) and studied in vivo under a light microscope (Leica DM2500, Leica Camera, Wetzlar, Germany). The rediae and metacercariae (n: 15) were studied and measured alive; measurements of cercariae (n: 15) were taken from heat-killed specimens. All measurements are provided in micrometers (μm) with the mean followed by the range in parentheses. Drawings were made with the aid of a drawing tube, and the photographs were made using the Leica Application Suite software ver. 4.1.0.1264. Some specimens (vouchers) were deposited at the Parasitological Collection (CNP-Par) of the Instituto de Biología de Organismos Marinos, CCT CONICET-CENPAT, Puerto Madryn, Chubut Province, Argentina. The prevalence of the parasite (P) was calculated as the number of infected snails divided by the total number of snails examined at each site, expressed as a percentage. The number of metacercariae per parasitized snail was counted, and the mean intensity was calculated as the total number of metacercariae divided by the number of parasitized snails (I). The intensity of infection is presented as the mean followed by the interval in parenthesis. The prevalence and intensity of metacercariae were calculated only by counting metacercariae outside the rediae (i.e., when cercariae leave the host and enter the same snail or another snail). 

### 2.2. Histology

After emission observations, all specimens were dissected to see if they had pre-patent infections, measured with a caliper from the apex to the end of the spire, and subsequently processed according to the OIE-recommended protocol (https://www.oie.int/fileadmin/Home/eng/Health_standards/aahm/current/chapitre_general_information_2_4.pdf, accessed on 15 January 2022), including freezer chilling (−20 °C) for 10 min before dissection. A total of 98 specimens from both sites (R = 31; LP = 67) were processed for histology. For each snail, two biopsy cassettes were used to include the following organs: gonad, digestive gland, inhalant siphon, foot, lung, kidney, stomach, gills, mantle, and albumen gland or penis. The cassettes were placed in Davidson’s fixative for 24 h and processed with classical histological procedures [22]. One 5 μm thick section from each block was cut and stained with hematoxylin and eosin (H&E). Histological sections were examined under a light microscope to study the infection sites and damage to tissues and organs. Photographs were taken with a Leica DFC280 digital camera and associated software. The intensity of infection was qualitatively classified as follows: light (characterized by few rediae mainly containing germinal balls and undeveloped cercariae), moderate (intermediate number of rediae containing developed and/or undeveloped cercariae), and high (numerous rediae mainly containing developed cercariae and sometimes metacercariae). This represents an assessment of the formation of the parasite infrapopulation based on a time scale from the introduction of miracidium to the complete colonization of the mollusc body by reproducing rediae.

### 2.3. DNA Extraction, Amplification, and Sequencing

Some rediae (N = 3) and metacercariae (N = 8) were fixed in 96% ethanol and then washed for 24 h in a TRIS (10 mM)–EDTA (1 mM) buffer solution. DNA was extracted using the GenEluteTM Mammalian Genomic DNA Miniprep Kit (Sigma, St. Louis, MO, USA) according to the manufacturer’s instructions. The 28S regions of the rDNA for the rediae and metacercariae were amplified by PCR. PCRs were performed in a total volume of 50 µL containing 10× buffer (200 mM Tris-HCl pH 8.4, 500 mM KCl), 0.2 mM of each dNTP, 1.5 mM of MgCl_2_, 0.4 µM of each primer, and 1 U of platinum Taq polymerase. In total, 2 µL of genomic DNA was used as a template. The 28S regions were amplified using 28S-28S: 5″-GTGAATACCCGCTGAACTTAAGC-3″ as a forward primer, situated 16 bp from the 3″ end of the conserved region of the lsrDNA, and 28S-28S: 5″-TCTCCTTGGTCCGTGTTTCAA-3″ as a reverse primer, located 868 bp from the 5″ end of the conserved region of the lsrDNA. These primers were used following sources such as Cremonte et al. [23]. The cycling conditions included an initial denaturation at 94 °C for 5 min, followed by 40 cycles of 30 s at 94 °C, 30 s at 52 °C (annealing), and 2 min at 72 °C, with a final extension step of 10 min at 72 °C. Amplified PCR products were electrophoretically separated in a 1 % (*w*/*v*) agarose gel stained with gel green. Negative controls for the PCR were always run to control for contamination. Relevant bands were sent for purification and sequencing (MacroGen, Seoul, Republic of Korea). All sequences have been deposited in GenBank. 

### 2.4. Sequence Alignments and Phylogenetic Analysis

The partial 28S rDNA sequences generated in this study were aligned with sequences of representatives of the Echinostomatidae family available in GenBank (Table 1) and *Fasciola hepatica* Linnaeus, 1758 (Fasciolidae) (AY222244), which was used as an outgroup. The concatenated alignments were performed using the MAFFT software ver. 7 (available at http://www.ebi.ac.uk/Tools/msa/mafft/, accessed on 23 February 2024) and MEGA X [24]. Phylogenetic and molecular evolutionary analyses were conducted on the aligned nucleotide sequences of 28S and were inferred by both the maximum likelihood (ML) method using the W-IQTREE 1.0 online software [25] and Bayesian inference (BI) using BEAST v1.8.0 [26]. For the ML tree, the best-fit model chosen according to the Akaike information criterion (AIC) was GTR + F + I + G4. Node supports were evaluated using a bootstrap ultrafast test with 1000 replicates [27]. The percentage of trees in which the associated taxa clustered together is shown next to the branches. For the BI tree, to determine the evolution model that best fits our dataset, the program jModeltest 2.1.1 [28] was employed, with model selection based on AIC. The results indicated that GTR + G was the most appropriate. Markov Chain Monte Carlo (MCMC) chains were run for 10,000 generations, sampling every 10 generations, with the first 250 sampled trees discarded as “burn-in”. Finally, a 50 % majority rule consensus tree was constructed. These analyses involved 43 nucleotide sequences with 995 positions in the final dataset. Genetic divergences (p-distance) were calculated for 19 nucleotide sequences grouped in the clade, which include the species studied here, using Mega X.

## 3. Results

Table 2 shows the apple snail data for shell length and sex ratio from the Harbor Natural Reserve (R) and Los Padres Lagoon (LP). Furthermore, the prevalence and intensity of infection for both stages found—rediae (with developing cercariae and/or metacercariae) and metacercariae—are given. The prevalence was higher in the Reserve than in Los Padres. Half of the infected snails in the Reserve had pre-patent infections (i.e., the snails did not shed cercariae), while the only infected snail in LP was emitting cercariae. Encysted metacercariae were found by stereoscopical examination in the pericardial cavities of the snails at the two studied sites (Table 2). Furthermore, encysted metacercariae were found by observing the histological sections in the albumen gland and kidney. The species studied herein were identified based on morphology and genetics as belonging to the Echinostomatidae family. Rediae, cercariae, and metacercariae from R and LP are very similar morphologically, but they differ in 28S rDNA sequences.

### 3.1. Description of Redia, Cercaria, and Metacercaria of Echinostomatidae gen. et sp.

Considering the morphological similarities observed between the intra-molluscan stages of the digeneans collected from both sampling sites and the highest prevalence found in the Reserve, descriptions of the development stages were made with samples collected at this site. 

Rediae (Figure 1A)

Body size: 2090 (1560–2600) long by 560 (490–620) wide. Muscular pharynx: 8.3 (7.8–8.8) long by 7.7 (6.0–10) wide. Esophagus opening into a sac-like caecum: 877 (850–1260) long. Tegumentary collar behind the pharynx. A pair of locomotory appendages near the posterior end of the body. Birth pore immediately posterior to the tegumentary collar. Mature rediae containing 1 to 3 developing cercariae or 1 to 3 metacercariae.

**Figure 1:**
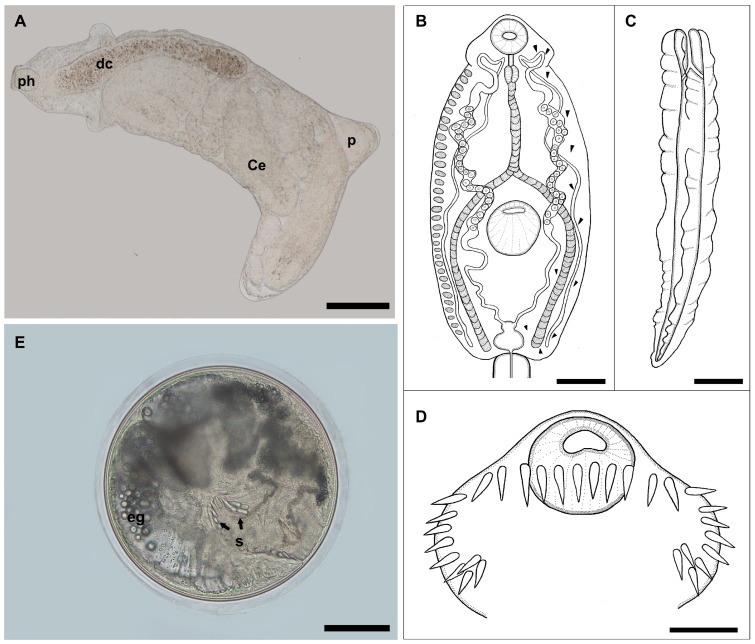
Developmental stages of Echinostomatidae gen. et. sp. (**A**) Redia, in vivo, showing pharynx (ph), caecum (dc), cercaria (Ce), podium (p); (**B**) cercarial body, line drawing; (**C**) tail ventral view, line drawing; (**D**) detail of head collar spines; (**E**) metacercaria from pericardial cavity, in vivo, showing the excretory granules (eg) and spines (s). Bars = 50 µm (**D**), 100 µm (**B**,**E**), 150 µm (**C**), and 200 µm (**A**).

Cercariae (Figure 1B–D) 

Body: 970 (750–1220) long by 420 (350–500) wide, without spines. Collar with 31 spines, with four corner spines on each side and a single anteriorly uninterrupted row of 23 spines. Oral sucker subterminal: 82 (79–90) long by 81 (67–90) wide. Ventral sucker: 127 (123–132) long by 131 (120–150) wide. Prepharynx: 35 (32–39) long. Pharynx: 45 (39–50) long by 39 (31–42) wide. Esophagus: 260 (200–290) long by 18 (15–24) wide, consisting of approximately 14 to 16 cells, bifurcates at 438 (336–480) from the anterior end of the body and caeca extending to the posterior end of the body. Numerous cystogenous cells are located between the pharynx and the posterior margin of the body. Excretory system stenostomate: The excretory vesicle was divided into two chambers located at the posterior end of the body. The primary excretory tubes originated in the anterior chamber of the excretory vesicle and dilated in the area between the ventral sucker and the pharynx to accommodate 21–30 spherical refractile granules 14 (11–18) in diameter. Flame cells were difficult to see, with at least 15 pairs. The caudal duct of the excretory system enters the anterior portion of the tail and bifurcates into two branches that end at the lateral margins of the tail. Tail: 960 (840–1200) long by 91 (70–120) wide, with four dorsoventral fin-folds, two anterior, two posterior, and one continuous lateral fin-fold that covers both sides of the tail.

Metacercariae (Figure 1E)

Metacercarial cysts are usually spherical and 336.6 (300–376.6) in diameter. Cyst wall: 11.3 (9–13).

*Taxonomic summary*

First and second intermediate hosts: *Pomacea canaliculata* (Lamarck, 1822) (Ampullariidae, Caenogastropoda).

Sites of infection: Rediae with cercariae and/or metacercaria in the gonad, digestive gland, albumen gland, gills, intestine, kidney, lung, and mantle; metacercariae (outside the rediae) mainly in the pericardial cavity but also in the albumen gland and kidneys.

GenBank accession numbers: PP390560 (rediae from the Reserve), PP391013 (metacercariae from the Reserve), PP391016 (rediae from Los Padres), and PP391021 (metacercariae from Los Padres).

Specimens deposited: Rediae with cercariae and metacercariae (CNP-Par 226) and metacercariae (CNP-Par 227) from the Reserve (ethanol fixed specimens).

*Taxonomic remarks*

According to Pinto and de Melo ([38]) and Damborenea et al. ([7]), two species of echinostomatids have been reported to parasitize *P. canaliculata* in South America: *Echinostoma parcespinosum* (in Argentina and Brazil) and *Dietziella egregia* (Dietz, 1909) (in Argentina). Both species have a similar number of spines on the collar, 31 to 33 (*E. parcespinosum*) and 31 (*D. egregia*), which agrees with the number of spines found on the collar of the cercaria described herein (31). However, only a description of the cercariae of *E. parcespinosum* has been available for morphological comparisons since the life cycle of this species was elucidated by Martorelli [21]. The rediae of this species found in the Reserve are larger and wider but morphologically similar to those of *E. parcespinosum*. The cercariae found in *P. canaliculata* are distinguished from the cercariae of *E. parcespinosum* by having a larger body (750–1220 vs. 820–960), slightly smaller oral and ventral suckers (67–90 vs. 80–100, and 120–150 vs. 170–180 in diameter, respectively) and in the number of fin-folds in the tail. The cercariae from the Reserve had four dorsoventral fin-folds and one continuous lateral fin-fold, while the cercariae of *E. parcespinosum* have “a tail surrounded by a thin and translucent fin”. Regarding the metacercariae, the diameter of the cysts of the two species differs slightly (330–350 vs. 300–376.6).

### 3.2. Molecular Analyses

**Figure 2:**
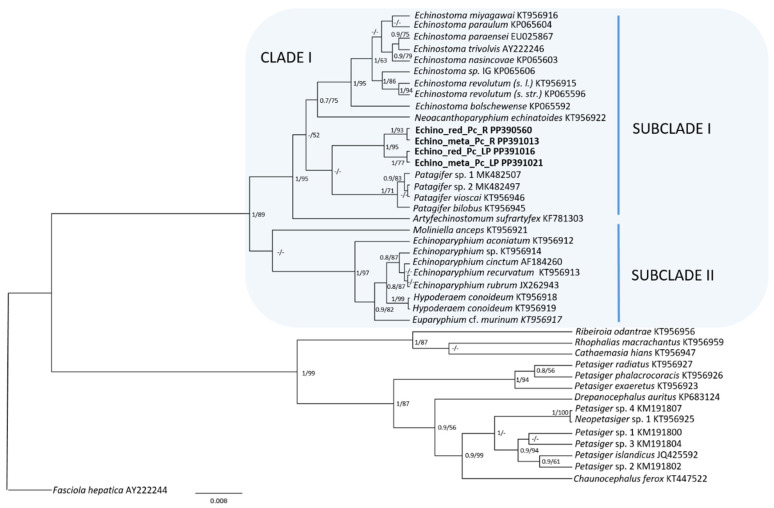
Phylogram resulting from using Bayesian inference (BI) and maximum likelihood (ML) on the partial 28S rDNA gene sequences of Echinostomatidae rooted in *Fasciola hepatica*. Posterior probability values (BI) and bootstrap values (ML) associated with the branches are shown as BI/ML; support values lower than 0.70 (BI) and 50 (ML) are not shown. The scale bar indicates the number of substitutions per site. Newly generated sequences are highlighted in bold.

A total of four sequences including a partial 28S of rDNA was obtained. Rediae and metacercariae from the Harbor Natural Reserve (R) provided products of 895 pb and 906 pb, respectively, and they were 100% identical. Rediae and metacercariae from Los Padres Lagoon (LP) provided products of 917 pb and 871 pb, respectively, and they were 100% identical too. Sequences from R differed by 7 pb compared with sequences from LP. Phylogram trees built with ML and BI provided similar topologies, and the BI analysis had higher nodal support than the ML analysis (Figure 2). Species of the Echinostomatidae family are grouped into two big clades; one of them includes the genera *Echinostoma* Rudolphi, 1809; *Neoacanthoparyphium* Yamaguti, 1958; *Patagifer* Dietz, 1909; *Artyfechinostomum* Lane, 1915; *Moliniella* Hübner, 1939; *Hypoderaeum* Dietz, 1909; and *Echinoparyphium* Dietz, 1909. These genera are separated into two subclades: subclade 1 includes *Echinostoma*, *Neoacanthoparyphium*, *Patagifer*, and *Artyfechinostomum*, and subclade 2 includes *Moliniella*, *Hypoderaeum*, and *Echinoparyphium*. The species studied here is closer to the *Patagifer* clade, which is a monophyletic clade that includes four species. However, it seems to be a different genus composed of two species (one from R and another from LP). The genetic distance between the sequences from R and LP is 0.006, while the genetic distance between *Patagifer* spp. is 0.001–0.002, and the genetic distance between *Echinostoma* spp. is 0.003–0.013 (Table 3).

### 3.3. Sites of Infection and Host Tissue Damage

When *P. canaliculata* acts as the first intermediate host, the rediae with developing cercariae inside are found mainly occupying the gonad and digestive gland but also the gills, intestine, albumen gland, lung, kidney, mantle border, and penis. Five of the eight infections studied by histology were of moderate or high intensity. In three cases, the intensity of infection was light, and in these cases, although the rediae occupied the same organs, they were considerably less abundant. A double digenean infection was found only twice; sporocysts belonging to another digenean species were observed only occupying the digestive gland, and the infection intensity caused by the echinostomatid was light. 

**Figure 3:**
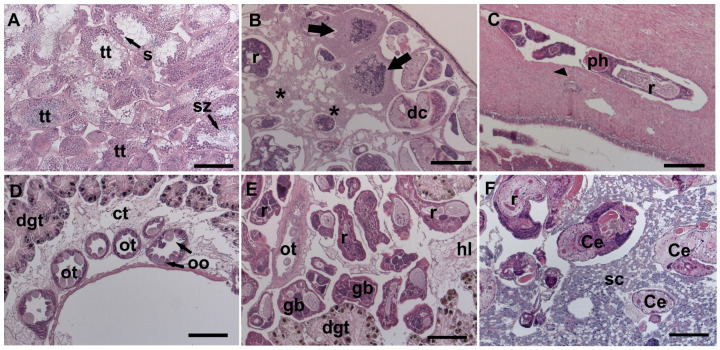
Histological sections (H&E) of unparasitized and parasitized snails. (**A**) Unparasitized testis. (**B**) Testis showing hemocytic encapsulation (black arrow) and tissue lysis (black asterisk). (**C**) Redia between the sheath groove of the penis (arrowhead). (**D**) Unparasitized ovary. (**E**) Atrophied ovarian tubule and gonadal connective tissue replaced by rediae in different stages of development. (**F**) Rediae with cercariae inside the albumen gland. Abbreviations: Ce (cercariae), dc (developing cercariae), dgt (digestive gland tubule), gb (germinal ball), hl (hemolymphatic space), oo (oocyte), ot (ovarian tubules), r (rediae), sc (secretory cells of the albumen gland), s (spermatocyte), sz (spermatozoa), and tt (testis tubules). Scale bars: 100 µm (**A**); 200 µm (**B**–**F**).

In uninfected snails, the male gonad occupies the first two spiral turns; in females, the ovary is a thread situated within the visceral mass at the inner apex of the spiral rim (Figure 3A,D). The partial or total castration of snails was observed in cases of moderate and high infection intensity, respectively. In the males, complete lysis of gonadal tissue and also hemocytic infiltration was observed due to the presence of rediae (Figure 3B). Furthermore, in one case, rediae were found in the penis sheath groove (Figure 3C). In light or moderate infection intensity, rediae were found in the connective tissue of the testis tubules; nevertheless, it was possible to observe the presence of some tubules. In females with high infection intensity, rediae invaded the connective tissues of the ovaries, which almost completely atrophied; however, no hemocytic infiltration was observed (Figure 3E). Moreover, in moderate or high infection intensity, rediae were also found in the secretory cells and ducts of the albumen gland (Figure 3F). 

**Figure 4:**
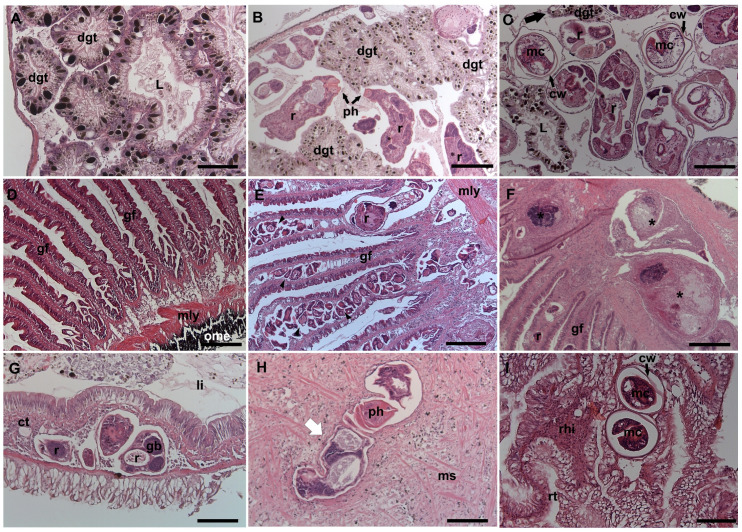
Histological sections (H&E) of unparasitized and parasitized snails. (**A**) Unparasitized digestive gland. (**B**) Rediae invading the connective tissue of the digestive gland. (**C**) Compressed digestive gland tube (black arrow) caused by rediae and metacercariae. (**D**) Normal structure of the gill filament. (**E**) Deformation of the gill filament caused by the presence of numerous rediae (black triangle). (**F**) Hemocytic encapsulation and granulocytomas (black asterisks) in the gill. (**G**) Enlargement of the intestinal wall caused by rediae in the connective tissue. (**H**) Rupture of the muscle fibers in the foot caused by the presence of rediae (white arrow). (**I**) Metacercariae between renal tubules. Abbreviations: cw (cyst wall), dgt (digestive gland tubules), gf (gill filament), hl (hemolymphatic space), L (lumen of digestive gland tube), li (light of the intestine), mly (muscle layer), ms (muscle fibers), ome (outer mantle epithelium), ph (pharynx), mc (metacercariae), r (rediae), c (cercariae), rhi (renal hemocyte islet), and rt (renal tubule). Scale bars: 100 µm (**A**,**G**,**H**,**I**); 200 µm (**B**–**F**).

In general, the presence of rediae was rarely found to alter the structure of the digestive gland tubules (Figure 4A), as they mostly occupied the connective tissue in the inter-tubular areas (Figure 4B). However, when infection intensity was high, a few tubules were deformed by compression (Figure 4C). No rediae were observed inside the tubules. The gills were, at all infection intensities, occupied by rediae, but structural damage was only observed in cases of high infection intensity (Figure 4D). In these cases, great deformations of the gill filament were observed due to the presence of rediae between the hemal sinuses and in the afferent arteries (Figure 4E). In addition, strong hemocytic infiltration was observed, resulting in the formation of granulocytomas around the parasite remnants (Figure 4F). The connective tissue of the intestine was occupied by rediae in most of the infection cases; when infection intensity was high, the organ was enlarged (Figure 4G). No significant damage was observed in the lung or kidney, although when rediae were found in the mantle or, unusually, in the foot or inhalant siphon, they destroyed muscle fibers (Figure 4H).

When *P. canaliculata* acted as the second intermediate host, most of the metacercariae were found infecting the pericardial cavity. Furthermore, by observing the histological sections, it was possible to find some metacercariae between the renal tubules and the ducts of the albumen gland (Figure 4I). They were typically spherical and thick-walled, and no host reaction was observed in any case.

## 4. Discussion

This work describes an echinostomatid digenean species in the apple snail taken from its native distribution area. The results of our molecular analyses suggest that, despite their morphological similarities, the echinostomatids found in the Reserve and Los Padres Lagoon belong to different species. Furthermore, analyses of the 28S sequences indicate that these species belong to a new genus close to *Patagifer* or a genus already described but without molecular data. Prevalence was higher in the Reserve, and this is probably due to the higher abundance of birds acting as definitive hosts. This is consistent with Lafferty et al. [39], Whitney et al. [40], and Etchegoin et al. [41], among others, who established that the diversity and abundance of birds were positively correlated with the richness and abundance of larval digeneans in snails.

In South America, the only published description of the intra-molluscan stages of echinostomatid parasitizing the apple snail belongs to the genus *Echinostoma* (*E. parcespinosum*) [21]. However, according to our molecular results, the digeneans studied here did not belong to this genus. Although it has not been described, another species parasitizing the apple snail has been recorded, belonging to the genus *Dietziella* Skrjabin et Bashkirova, 1956. In Argentina, Digiani [42] provided a description of an adult-stage *Dietziella egregia* (Dietz, 1909) obtained from the intestine of a white-faced ibis, *Plegadis chihi* Vieillot, in 1817 in Buenos Aires Province. The adults of this species have a collar of 31 spines in a single row, similar to that of the cercariae and metacercariae studied in this work. Furthermore, the metacercariae of *D. egregia* have been observed in the renal cavity of *P. canaliculata* from Argentina (Digiani and Ostrowski de Núñez, unpublished). To date, none of these records have shown molecular sequences.

We corroborated that *P. canaliculata* acts as an intermediate host of a species of echinostomatid; furthermore, we found that its life cycle can be abbreviated. It occurs when the same snail specimen acts as the first and second intermediate host (i.e., the cercariae encyst as metacercariae inside the rediae). Although this life cycle has not been elucidated, the definitive host must be an avian that feeds on the apple snail. In this sense, Bertolero and Navarro [43] highlighted the importance of *P. canaliculata* in the diet of the glossy ibis in Spain. Therefore, the white-faced ibis and *P. canaliculata* could be the potential hosts for *D. egregia* in Argentina. In any case, to link the intra-molluscan stages found in this work, genetic studies of the adult stage described (i.e., *D. egregia*) from the bird host should be carried out. Likewise, new molecular studies considering other regions of rDNA (ITS; nad1) and mDNA (cox1) are necessary to confirm the existence of two different species belonging to a unique genus closer to the *Patagifer* clade. 

According to Lauckner [44], the gonad is usually the primary organ infected by digenean larvae. As infection takes place in the hemocelic space of the gonad [19], the intra-molluscan stage often migrates to other organs of the host, such as the digestive gland [10,45]. In snails, both organs provide a continuous supply of energy [46] and play a vital role in facilitating growth and multiplication. This is why these organs are usually invaded; most digenean infections are reported in these sites of infection [19]. In some cases, when the infection intensity is high, the intra-molluscan stages invade other organs besides the gonad and digestive gland [14,47]. Nevertheless, here, we found that, even in recent infections (i.e., light intensity), the echinostomatid invades several snail organs (i.e., the gills, intestine, albumen gland, lung, kidney, and mantle border), not only the gonad and the digestive gland, as is usual in digeneans. Therefore, we can conclude that the degree of damage and the invaded organs depends not only on the infection intensity but also on the abundance and size of the rediae. Although it is common to find more than one parasite species in the same host species (e.g., [48]), in this work, only two cases of double infection were found. In this sense, it was observed that (i) the infection by the echinostomatid was recent and restricted the development of the other parasite in the digestive gland (where almost no rediae were observed), and (ii) the double infection did not result in a higher pathogenicity than the single infection, as observed by Gilardoni et al. [14]. As mentioned by Galaktionov et al. [9], rediae are also capable of consuming the sporocysts of other trematode species, as well as their own juvenile specimens. Therefore, the coexistence of echinostomatids with sporocyst species in molluscs seems to occur mainly in cases of recent echinostomatid infections.

Histologically, the gonads of uninfected snails possessed abundant mature gametes in both sexes. In males, Sertoli cells form a continuous sheath around each testis tubule, whereas in females, ovarian tubules are sparse, but numerous oogenic cells lie directly on the basal membrane [49]. When compared with infected gonads, histopathological changes were significant. The changes and damage observed in the present work suggest that the echinostomatid can cause complete castration in the host when the infection intensity is high. Parasitic castration in molluscs has been widely reported [12,19,50]. Lafferty and Kuris [51] indicated that parasitic castration involves mechanical and physiological damage. This damage initially affects the gonads and subsequently alters the overall physiological balance of the host. Our work suggests that, in males, parasitic castration seems to be associated with physiological damage since loss of tissue integrity and necrosis or lysis of the testis tubules were observed. In females, instead, this phenomenon seems to be related to mechanical damage. In cases of high infection intensity, the structure of the ovarian tubules was altered by the replacement of tissues with rediae. In cases of light or moderate infection intensity, remnants of the ovarian tubules were observed, although in a reduced condition, with fewer and atrophied oocytes. It is likely that the snail–digenean relationship studied here also has physiological effects that cause castration, as demonstrated by Averbuj and Cremonte [11]. However, in addition to the gonad, the digestive gland is usually one of the first organs to be invaded by digeneans; in most of the cases examined in this study, the rediae were in the inter-tubular areas of the gland and rarely altered its structure. In light infections, the digestive tubules were either normal or more widely spaced; in moderate or high infections, a few tubules were deformed by compression. This contrasts with findings in previous works, where the presence of digeneans caused more damage to the digestive gland (e.g., [10,45]).

When rediae were found in the gills, the degree to which they damaged the organ could be correlated with the infection intensity. In high infections, the gill filaments were deformed, which could imply impaired respiratory functions. Although there is no record of this, Laurelle et al. [52] found, in zebra mussels, that there are digeneans that only infect the gills and alter their structure. Ampullarid snails are well adapted to three different modes of respiration: gill respiration, siphonal pulmonary respiration, and direct pulmonary respiration [53]. In particular, gill respiration is achieved while the snails are submerged in a continuous flow of water within the mantle cavity [53,54]. Since snails remain submerged for most of the year, the presence of rediae in the gills could negatively affect snail survival. So far, there are no studies that prove the presence of rediae in the gills has a direct effect on host mortality. Additionally, occasional signs of inflammation and hemocytic infiltration were observed, leading to the formation of granulocytomas. This strong host response appears to be atypical in molluscs that act as first intermediate hosts for digenean trematodes [47]. However, this reaction may indicate that *P. canaliculata* has the immunological capacity to recognize foreign objects (see [55]) such as digenean trematodes. Further studies are still required to understand how the severity of parasitic infection affects the health of snails, for example, by assessing oxygen consumption or the RNA/DNA ratio. These methods have been shown to be reliable and useful as indicators of health status [56].

Brito, et al. [1] suggested that several options have been presented for the control of *P. canaliculata*, such as biological and chemical control. Parasitic castration emerges as a natural and highly effective method of regulating this species since, at the individual level, it is detrimental, while at the population level, it will depend on the prevalence [57]. This could have a significant role in controlling density and preventing the overpopulation of this species.

## 5. Conclusions

This study showed that rediae and metacercariae (existing outside the rediae, separately in other organs) found in the snail *Pomacea canaliculata* belonged to the family Echinostomatidae. Molecular analyses revealed that they could be intra-molluscan stages of species included in a genus already described (i.e., *Dietziella*) or in another that has not yet been described related to the genus *Patagifer*. Regarding the life cycle, it was established that digeneans used *P*. *canaliculata* both as first and second intermediate hosts. Although the intra-molluscan stages of the parasites were morphologically very similar between sampling localities, the sequences differed by 7 bp, which suggests that they may be two different species. Finally, histological analysis showed that the parasites had a detrimental effect on the snail, causing damage to many organs and complete castration.

## Figures and Tables

**Table 1:** Summary data for 28S rDNA sequences of echinostomatoid taxa included in the molecular analyses. Abbreviations: red (redia), cer (cercaria), meta (metacercaria), ad (adult), Pc (*Pomacea canaliculata*), R (Reserve), and LP (Los Padres).

Taxon	Life Cycle Stage	Host	Country	GenBank N°	References
**Family Echinostomatidae Looss, 1899**					
Subfamily Echinostomatinae Looss, 1899					
Echinostomatidae gen. et sp. (**red_Pc_R**)	red	*Pomacea canaliculata* (Lamarck)	Argentina	PP390560	Present study
Echinostomatidae gen. et sp. (**meta_Pc_R**)	meta	*Pomacea canaliculata*	Argentina	PP391013	Present study
Echinostomatidae gen. et sp. (**red_Pc_LP**)	red	*Pomacea canaliculata*	Argentina	PP391016	Present study
Echinostomatidae gen. et sp. (**meta_Pc_LP**)	meta	*Pomacea canaliculata*	Argentina	PP391021	Present study
*Echinostoma miyagawai* Ishii, 1932	ad	*Anas platyrhynchos* L.	Ukraine	KT956916	[29]
*Echinostoma paraulum* Dietz, 1909	cer	*Lymnaea stagnalis* (L.)	Germany	KP065604	[30]
*Echinostoma paraensei* Lie and Basch, 1967	ad	“hamster”	USA	EU025867	[31]
*Echinostoma trivolvis* (Cort, 1914)	ad	*Mesocricetus auratus* Waterhouse (exp.)	USA	AY222246	[32]
*Echinostoma nasincovae* Faltýnková et al., 2015	cer	*Planorbarius corneus* (L.)	Czech Republic	KP065603	[30]
*Echinostoma* sp. IG	cer	*Radix auricularia* (L.)	Germany	KP065606	[30]
*Echinostoma revolutum* (Frölich, 1802) (*sensu lato*)	ad	*Aythya collaris* (Donovan)	USA	KT956915	[29]
*Echinostoma revolutum* (Frölich, 1802) (sensu stricto)	ad	*Aythya fuligula* (L.)	Czech Republic	KP065596	[30]
*Echinostoma bolschewense* (Kotova, 1939)	cer	*Viviparus acerosus* (Bourguignat)	Slovakia	KP065592	[30]
*Neoacanthoparyphium echinatoides* (de Filippi, 1854)	cer	*Viviparus acerosus* (Bourguignat)	Slovakia	KT956922	[29]
*Moliniella anceps* (Molin, 1859)	meta	*Planorbarius corneus* (L.)	Lithuania	KT956921	[29]
*Echinoparyphium aconiatum* Dietz, 1909	cer	*Lymnaea stagnalis* (L.)	Czech Republic	KT956912	[29]
*Echinoparyphium* sp.	ad	*Anas clypeata* L.	USA	KT956914	[29]
*Echinoparyphium cinctum* (Rudolphi, 1803)	ad	*Anas platyrhynchos* L.	Ukraine	AF184260	[33]
*Echinoparyphium recurvatum* (von Linstow, 1873)	cer	*Radix ovata* (Draparnaud)	Slovakia	KT956913	[29]
*Echinoparyphium rubrum* Cort, 1914	cer	*Planorbella trivolvis* (Say)	USA	JX262943	[34]
*Hypoderaeum conoideum* (Bloch, 1782)	ad	*Anas platyrhynchos* L.	Ukraine	KT956918	[29]
*Hypoderaeum conoideum* (Bloch, 1782)	ad	*Anas acuta* L.	USA	KT956919	[29]
*Euparyphium cf. Murinum* Tubangui, 1931	ad	*Malacomys longipes* Milne-Edwards	Uganda	KT956917	[29]
*Petasiger radiatum* Dujardin, 1845	ad	*Phalacrocorax carbo* (L.)	Ukraine	KT956927	[29]
*Petasiger phalacrocoracis* (Yamaguti, 1939)	ad	*Phalacrocorax carbo* (L.)	Ukraine	KT956926	[29]
*Petasiger exaeretus* Dietz, 1909	ad	*Phalacrocorax carbo* (L.)	Ukraine	KT956923	[29]
*Drepanocephalus auritus* Kudlai et al., 2015	ad	*Phalacrocorax auritus* (Lesson)	USA	KP683124	[35]
*Petasiger* sp. 1	cer	*Planorbis planorbis* (L.)	Czech Republic	KM191800	[36]
*Petasiger* sp. 2	cer	*Gyraulus albus* (O. F. Müller)	Germany	KM191802	[36]
*Petasiger* sp. 3	cer	*Planorbis planorbis* (L.)	Germany	KM191804	[36]
*Petasiger* sp. 4	meta	*Gasterosteus aculeatus* L.	Canada	KM191807	[36]
*Neopetasiger* n. sp.	ad	*Podiceps grisegena* (Boddaert)	USA	KT956925	[29]
*Petasiger islandicus* Kostadinova and Skírnisson, 2007	ad	*Aechmophorus occidentalis* (Lawrence)	USA	KT956924	[29]
Subfamily Nephrostominae Mendheim, 1943					
*Patagifer* sp. 1	cer	*Biomphalaria sudanica* (Marlens)	Kenia	MK482507	[37]
*Patagifer* sp. 2	cer	*Biomphalaria pfeifferi*	Kenia	MK482497	[37]
*Patagifer vioscai* Lumsden, 1962	ad	*Eudocimus albus* (L.)	USA	KT956946	[29]
*Patagifer bilobus* (Rudolphi, 1819)	ad	*Plegadis falcinellus* (L.)	Ukraine	KT956945	[29]
Subfamily Himasthlinae Odhner, 1910					
*Artyfechinostomum sufrartyfex* Lane, 1915	ad	*Sus scrofa* dom.	India	KF781303	-
Subfamily Chaunocephalinae Travassos, 1922					
*Chaunocephalus ferox* (Rudolphi, 1795)	ad	*Ciconia nigra* (L.)	Ukraine	KT447522	-
**Family Psilostomidae Looss, 1900**					
Subfamily Ribeiroiinae Travassos, 1951					
*Ribeiroia ondatrae* (Price, 1931)	ad	*Pelecanus erythrorhynchos* Gmelin	USA	KT956956	[29]
**Family Cathaemasiidae Fuhrmann, 1928**					
Subfamily Cathaemasiinae Fuhrmann, 1928					
*Cathaemasia hians* (Rudolphi, 1809)	cer	*Planorbis planorbis* (L.)	Czech Republic	KT956947	[29]
**Family Rhopaliidae Looss, 1899**					
*Rhopalias macracanthus* Chandler, 1932	ad	*Didelphis virginiana* (Kerr)	USA	KT956959	[29]
**Family Fasciolidae Railliet, 1895**					
Subfamily Fasciolinae Railliet, 1895					
*Fasciola hepatica* Linnaeus, 1758	ad	*Capra hircus* (L.)	Saudi Arabia	AY222244	[32]

**Table 2:** Apple snail data (shell length and sex ratio) from the two sampling sites in Argentina with prevalence and mean intensity of infection caused by the digenean trematode Echinostomatidae gen. et. sp. Prevalence and mean intensity are followed by range in parenthesis. The first part of the table shows the results for the total number of snails collected after stereomicroscopic examination; the second part includes the results for the subsample processed for histology and examined under a light microscope.

Sampling Sites	Harbor Natural Reserve	Los Padres Lagoon
N total (stereoscope microscope examination)	67	137
Shell length mean (±SD) (cm)	4.37 (0.7)	4.87 (0.7)
Sex ratio (female/male)	41/25	83/54
Prevalence of rediae (%)	15.1	0.72
Prevalence of metacercariae (%)	74.2	9.5
Mean intensity of metacercariae	298 (17−1912)	41 (2−169)
N (histology subsample)	31	67
Shell length mean (±SD) (cm)	4.34 (0.8)	4.86 (0.8)
Sex ratio (female/male)	19/31	41/67

**Table 3:** P-distance of 28S sequences of 19 species belonging to subclade 1 in the phylogenetic tree built for this study. 1. Echino_red_Pc_R; 2. Echino_meta_Pc_R; 3. Echino_red_Pc_LP; 4. Echino_meta_Pc_LP; 5. *Patagifer* sp. 1; 6. *Patagifer* sp. 2; 7. *Patagifer bilobus*; 8. *Patagifer vioscai*; 9. *Echinostoma nasincovae*; 10. *Echinostoma miyagawai*; 11. *Echinostoma revolutum* (sl); 12. *Echinostoma trivolvis*; 13. *Echinostoma paraensei*; 14. *Echinostoma paraulum*; 15. *Echinostoma revolutum* (sr); 16. *Echinostoma bolschewense*; 17. *Echinostoma* sp. IG; 18. *Artyfechinostomum sufrartyfex*; 19. *Neoacanthoparyphium echinatoide*.

	1	2	3	4	5	6	7	8	9	10	11	12	13	14	15	16	17	18
1.																		
**2.**	**0.00000**																	
3.	0.00600	**0.00490**																
4.	0.00600	0.00490	**0.00000**															
5.	0.01918	0.01838	0.01542	**0.01501**														
6.	0.01799	0.01716	0.01423	0.01386	**0.00115**													
7.	0.01679	0.01593	0.01305	0.01270	0.00231	**0.00115**												
8.	0.01799	0.01716	0.01423	0.01386	0.00115	0.00000	**0.00115**											
9.	0.01559	0.01593	0.01661	0.01617	0.02076	0.01961	0.01845	**0.01961**										
10.	0.01799	0.01838	0.01898	0.01848	0.02307	0.02191	0.02076	0.02191	**0.00461**									
11.	0.02638	0.02574	0.02728	0.02656	0.03114	0.02999	0.02884	0.02999	0.01269	**0.01153**								
12.	0.02038	0.02083	0.02135	0.02194	0.02653	0.02537	0.02422	0.02537	0.00577	0.00807	**0.01615**							
13.	0.01918	0.01961	0.02017	0.01963	0.02422	0.02307	0.02191	0.02307	0.00346	0.00577	0.01153	**0.00461**						
14.	0.01687	0.01724	0.01788	0.01740	0.02202	0.02086	0.01970	0.02086	0.00348	0.00348	0.01159	0.00695	**0.00463**					
15.	0.02558	0.02491	0.02651	0.02579	0.03044	0.02927	0.02810	0.02927	0.01171	0.01054	0.00468	0.01522	0.01288	**0.01054**				
16.	0.02038	0.02083	0.02017	0.01963	0.02307	0.02191	0.02076	0.02191	0.01269	0.01038	0.02076	0.01384	0.01153	0.01159	**0.01991**			
17.	0.02158	0.02206	0.02254	0.02194	0.02653	0.02537	0.02422	0.02537	0.01038	0.00923	0.01384	0.01384	0.01153	0.00927	0.01288	**0.01499**		
18.	0.03234	0.03182	0.03081	0.02999	0.03114	0.02999	0.02884	0.02999	0.03345	0.03345	0.03922	0.03691	0.03460	0.03244	0.03864	0.03460	**0.03460**	
19.	0.02278	0.02328	0.02135	0.02079	0.02540	0.02425	0.02309	0.02425	0.02425	0.02425	0.02771	0.02771	0.02309	0.02320	0.02931	0.02079	0.02656	0.03460

## Data Availability

The molecular data will be available on GenBank (https://www.ncbi.nlm.nih.gov/nucleotide/, accessed on 28 January 2024), and parasite specimens will be deposited in the Parasitological Collection IBIOMAR CCT CONICET-CENPAT.
